# Four-state ferroelectric spin-valve

**DOI:** 10.1038/srep09749

**Published:** 2015-05-11

**Authors:** Andy Quindeau, Ignasi Fina, Xavi Marti, Geanina Apachitei, Pilar Ferrer, Chris Nicklin, Eckhard Pippel, Dietrich Hesse, Marin Alexe

**Affiliations:** 1 Max Planck Institute of Microstructure Physics, Halle, D-06120, Germany; 2 Department of Physics, University of Warwick, Coventry, CV4 7AL, United Kingdom; 3 Institute of Physics ASCR, v.v.i., Cukrovarnická 10, Praha 6, 162 53, Czech Republic,; 4 Centre d’Investigacions en Nanociencia i Nanotechnologia (ICN2), CSIC-ICN, Bellaterra, Barcelona, 08193, Spain; 5 Diamond Light Source, Harwell Science and Innovation Campus, Chilton, Didcot OX11 0DE, United Kingdom

## Abstract

Spin-valves had empowered the giant magnetoresistance (GMR) devices to have memory. The insertion of thin antiferromagnetic (AFM) films allowed two stable magnetic field-induced switchable resistance states persisting in remanence. In this letter, we show that, without the deliberate introduction of such an AFM layer, this functionality is transferred to multiferroic tunnel junctions (MFTJ) allowing us to create a four-state resistive memory device. We observed that the ferroelectric/ferromagnetic interface plays a crucial role in the stabilization of the exchange bias, which ultimately leads to four robust electro tunnel electro resistance (TER) and tunnel magneto resistance (TMR) states in the junction.

Originally conceived as a sensing technology, the discovery of the GMR effect^1^,^2^,^3^ quickly attracted plenty of attention because of its potential to revolutionize the memory market^4^,^5^,^6^,^7^. While the original design presented no memory and was insensitive to the sign of an applied magnetic field, the insertion of an adjacent AFM layer delivered hysteresis and hence the factual two-state memory^4^. Over two decades after discovering the GMR, AFM layers continue to be an indispensable component for the pinning of the magnetic moment, e.g. in magnetoresistive random access memories (MRAM) based on magnetic tunnel junctions (MTJ)^8^,^9^, despite of tremendous progress in other aspects such as the means for writing and reading magnetic memories. In the subsequent years until today, much effort has been dedicated into the design of novel architectures with multiple magnetic and electric stable resistance states^10^,^11^,^12^, which would be a significant upgrade of earlier conceived^13^ and realized^14^,^15^ multi-bit cell memories.

In this regard, multiferroic (MF) tunnel junctions, based on MTJ with replacement of the insulating layer by a ferroelectric one, have demonstrated the ability to induce long-range magnetic ordering in the ferroelectric (FE) layer^16^, to tune the spin-polarization of the top electrode by electric fields^17^, to manipulate the magnetism in an interfacial oxide layer^18^; and to switch between distinct tunnel magneto-resistance values (in sign and magnitude) as a function of the polarization of the FE layer, among others^19^. Despite the progress with multiferroics, which included the realization of four different magnetically and electrically independent states^20^,^21^,^22^, the integration of spintronic functionalities in a multiferroic system has remained an open task so far.

In this letter, we engineer a spin valve without auxiliary AFM layers while preserving the memory functionality. We show that the ferroelectric/ferromagnetic (FE/FM) interface in multiferroic tunnel junctions is responsible for the observed effects. As well as reproducing the previously reported coupling between tunnel electro-resistance^23^ and tunnel magneto-resistance^24^, we demonstrate that the sign of the unidirectional magnetic anisotropy ultimately allows a four-resistance state memory.

While TER naturally delivers two stable resistance states due to the polarization-induced tuning of the effective tunnel barrier height^23^,^25^, a source for unidirectional magnetic anisotropy is required to achieve true memory functionality in MTJs. This function could be accomplished by the introduction of an AFM layer close to either one of the ferromagnetic electrodes in the MFTJ. However, the recent discovery of magnetic polarization of the FE located at the interfaces^16^ enabled the engineering of the proper coupling between this metamagnetic layer in the FE barrier and the neighboring FM electrode. In the present work, we have prepared MFTJs with a variety of interface chemical compositions in order to tune the strength of the interfacial magnetic interactions. In this way we mimic the spin-valve response by producing an effective exchange bias (EB)^26^,^27^ at the multiferroic interface without inserting any intentional AFM layer.

The main results are summarized in Fig. 1. We have prepared a MFTJ comprising La_0.7_Sr_0.3_MnO_3_ (LSMO), PbTiO_3_ (PTO) and cobalt with layer-thicknesses of 20, 3.2, and 40 nm, respectively. We recall that there are no auxiliary AFM layers in the heterostructure. Synchrotron-based X-ray diffraction including Kiessig fringes from the ferroelectric barrier (Fig. 1a) and scanning transmission electron microscopy (STEM) images (Fig. 1b) confirm the high quality of the fully coherent epitaxial stack with sharp interfaces. Piezoresponse force microscopy (PFM) characterization proves the ferroelectric nature of the very thin PTO layer (see Supplementary Information). The as-grown multilayer stack was patterned by a wet etching process, with which junctions of 1600 μm^2^ area were realized. Conventional TMR signals with amplitudes of ~30% are obtained and shown in Fig. 1c, where a strong asymmetry in each curve can be observed. Remarkably, Co and LSMO coercive fields are in accordance with the ones determined from the TMR loops (see Supplementary Information). The two data sets correspond to two opposite directions of the applied magnetic field during field-cooling (FC) with a saturating in-plane magnetic field of 800 mT down to a base temperature of 5 K. We thus observe a clear signature of unidirectional magnetic anisotropy selected via the direction of the magnetic field during the FC. It is most relevant that the induced unidirectional anisotropy is strong enough as to deliver two distinct resistance states at a zero applied magnetic field, without the requirement of any AFM layer as in the archetypical MTJ memory devices^8^. Quantitatively up to 8% separation in the remanent state of the resistance is observed, which exceeds the lower bound of ~0.5% displayed by the earliest commercial magnetic random access memories (RAMs) by an order of magnitude^28^. The observed features persist at least tens of repetitions (Fig. 1d) with no empirically detected obstacle for further recurrences, and without significant decrease of the observed exchange bias. The observed memory effect in TMR can be combined with the TER effect. By using 5 μs long voltage pulses of amplitude±3 V, we can toggle between two ~300% separated TER states, which are set independently from the magnetic states. Therefore we have demonstrated a genuine 4-state memory concept device (Fig. 1e) originating from an interface effect, as we will discuss in detail in the following paragraphs.

In consistence with the preceding literature, we have observed that cobalt is a necessary actor for the effect to occur. In identically prepared devices but with a non-magnetic copper layer as the top-most electrode, we have observed the tunnel anisotropic magnetoresistance effect between LSMO and Cu (see Supplementary Information), without the observation of sizeable magnetic anisotropy. The experiments probe the change in the density of states at the Fermi energy (EF) of the LSMO and render a symmetric signal with respect to the externally applied magnetic field. Hence, the induced exchange bias-like unidirectional anisotropy (Fig. 1c) unambiguously stems from the FE/Co interface.

Having recognized that the effect originates from the topmost interface, we have prepared three samples with distinct interfaces in order to further investigate the role of the ferroelectric-Co interface. In Fig 2a,b,c, we present them in a series with increasing Zr/Ti ratio at the interface (samples in which PTO has been replaced by Pb_0.2_Zr_0.8_TiO_3_ (PZT) as depicted in Fig. 2b, and by PTO/PbZrO_3_ (PZO) bilayer as depicted in Fig 2c). The similar good quality of the three interfaces can be inferred from the TEM images. The imaging conditions of Fig. 2e reveal the interface-parallel atomic planes of the cobalt electrode, whereas the latter are not visible in Fig. 2d,f due to slightly different imaging conditions, although some texture might still exist in these latter cases.

In Fig. 2d–f, the measured TMR signals, obtained as described for the data in Fig 1, are plotted for both ferroelectric polarization directions and the whole set of samples (ferroelectric properties of all samples are shown in Supplementary Information). It can be observed that by increasing the Zr concentration (compare Fig. 2d,e), both the magnetic coercive field and the unidirectional anisotropy of the Co FM electrode decrease. The extreme case is that one, where one monolayer of PZO is introduced in between the PTO and Co layers (Fig. 2f). Here the unidirectional anisotropy vanishes. The chemically driven modification of unidirectional anisotropy seems to be independent of the trendless amplitude of the TMR shown in Fig. 2(d–f), which probes the spin-polarization at the top electrode of the tunnel junction and is constant for all samples. Therefore, the band structure around the Fermi energy of the ferromagnetic metal is rather interface-independent, hindering an eventual chemical variance among the samples. In contrast, the strength of the magnetic interface pinning is interface-dependent and it can be tuned by the B-site chemical composition of the last perovskite layer at the Co-FE interface. Note also that the LSMO magnetic coercive field remains broad and ill-defined for the three samples.

We now address the temperature dependence of the observed phenomena. Several TMR loops were collected at distinct temperatures following the same FC procedure (Fig. 3a). The data show that the exchange bias is stronger at low temperatures and decreases upon heating, vanishing at around 140 K (Fig. 3b). A detailed inspection of Fig 3c reveals that the amplitude of the TMR is simultaneously reduced by a factor ~2 thus evidencing a strong mutual interplay between the metamagnetic pinning layer and the reduction of the interface magnetization with the concomitant variation on density of states (DOS) of the top FM electrode.

In between the Zr- and the Ti-only terminated insulating layers, there is one interesting composition that we revisit here. By occupying the B-site with Zr_0.2_Ti_0.8_, Pantel *et al.*^19^ reported a sign change of the TMR, which can be easily obtained by chemical means.^29^, but there explained as a magnetoelectric swapping of the roles of majority and minority spins at the top electrode. Here, we have reproduced the former results in Fig 2h,k, now emphasizing the discussion on the sign of the interfacial unidirectional magnetic anisotropy. In our experiments, as expected for this intermediate stoichiometry, the magnetic pinning features (i.e. the asymmetry of the curve) are present but weaker than in the Ti-only interface and a weak unbalance of the magnetic asymmetry sign is visible. Therefore, we evidenced that i) magnetic anisotropy is sensitive to the ferroelectric switching and, as reported previously^19^,^24^, the bandstructure is dependent on the FE electric polarization, and that ii) the polarization sign affects the interface magnetic anisotropy revealing the presence of magnetoelectric response at the interface. Note also that the magnetic coercivity does not change upon ferroelectric switching (see Fig 2d), indicating that the ferroelectric switching only leads to a change of the magnetic interfacial coupling and not to a change of the intrinsic magnetic properties of Co.

Fitting of the I(V) characteristics shown in Fig 3e using the Brinkman^30^ model renders very similar work functions at the FE/Co interface for opposite polarization directions (Fig. 3d) and suggests that the observed TER stems from the LSMO/FE interface. In all, the FE switching does not significantly change either the interfacial magnetic anisotropy or the charge carrier density at the interface. Instead, the experimental results converge to the theoretical predictions in which the picometric Zr/Ti displacement within the unit cell enables to tune the DOS of the FM in front of the tunnel barrier.

The aforementioned observations indicate that the two FM and FE materials separated by the interface cannot be treated as separate entities and involve a very strong mutual interdependence. This is an important point towards discussing the role of the virtually unavoidable metal oxidation at the interface, which can be at the origin of the observed magnetic anisotropy^31^. It is worth noting that while increasing the proportion of Zr at the interface (which tends to get more oxidized than Ti), the CoO layer can be gradually reduced explaining the observed reduction of magnetic anisotropy. On the other hand, the found big discrepancy between the observed blocking temperature (near 140 K) and the CoO Neel temperature (291 K) and the fact that the field cool process has been performed from 150 K (CoO Neel temperature) invokes a more complex scenario despite it cannot be ruled out. Very recently, it has been suggested that iron oxide forming at Fe/BaTiO_3_ interfaces presents ferromagnetic or antiferromagnetic ordering as a function of the FE polarization sign^18^. If the latter scenario would be the one applicable here, the magnetic anisotropy status upon FE switching in samples with Zr_0.2_Ti_0.8_, and after several FE commutations in those with Ti, would clearly be in contradiction. Instead, the observed magnetic pinning effect can be ascribed to the antiferromagnetic coupling between Ti and Co, as already predicted to be present at the interface^16^, or in the first monolayers of the magnetic electrode^32^. The interfacial antiferromagnetic long-range ordering in Ti-rich interfaces would be in agreement with the results presented here.

In conclusion, we have shown that proper FM/FE interfaces deliver similar functionalities as AFM layers in spin valves, namely the source of a unidirectional magnetic anisotropy selectable by magnetic field-cooling. The presence of this anisotropy allows the reading of two distinct resistance states after application of suitable external magnetic field. By exploiting the synergies with the TER, up to four distinct memory states readable by low voltage have been recurrently demonstrated. By studying the B-site stoichiometry in the Pb(Zr/Ti)O_3_ perovskite, we identified Ti-rich interfaces as a key contributor to tune the strength of such magnetic anisotropy. By scrutinizing the top electrode nature, the Zr content at the interface, and the temperature dependence of the TMR, we have observed signatures of a strong magnetic coupling between Ti and Co.

## Experimental Section

To realize multiferroic heterostructures containing magnetic LSMO, ferroelectric PTO and PZT and the antiferroelectric PZO, we used reflection high-energy electron diffraction (RHEED)-controlled pulsed laser deposition (PLD). All structures were grown on (001)-oriented TiO_2_-terminated SrTiO_3_ (STO) substrates^33^. In case of the 20 nm thick LSMO bottom electrodes, RHEED oscillations were observed to control the thickness using a laser fluence of 1 J cm^−2^, repetition rate of 1 Hz, 600 °C substrate temperature and an oxygen pressure of 0.15 mbar. For the antiferroelectric and ferroelectric ultrathin film barrier, the growth parameters were changed to 0.2 mbar and 0.28 mbar of oxygen pressure and to laser fluencies of 0.5 J cm^−2^ and 1 J cm^−2^ for PTO/PZT and PZO, respectively. The ablation frequencies were in the range of 4 to 10 Hz, depending on the terrace width of the substrates. Magnetron sputtering was performed to deposit the cobalt electrodes in a 2.5·10^−3^ mbar Ar atmosphere, whereas gold capping layers and copper electrodes were deposited via thermal evaporation from a tungsten boat. The MFTJs were patterned by a wet etching process using a diluted potassium iodide based etchant after UV light lithography to produce capacitors of 1600 μm^2^ area.

All tunnel barriers of the discussed junctions are in the same thickness region of about 8 unit cells or 3.2 nm. This was ensured by the RHEED oscillation observation for the ferroelectric thin films (see Supplementary Information). The morphology of all ferroelectrics, investigated by AFM imaging, show characteristics of layer-by-layer growth and resemble the widths and heights of the underlying STO step terraces. The cobalt top electrode was grown mostly epitaxially textured with respect to the ferroelectric oxide film in case of PTO, which is representative for the other compositions containing cobalt (see Supplementary Information).

Surface X-ray diffraction (SXRD) data were recorded at beamline I07 of the Diamond Light Source, using 20 keV x-rays and a large six-circle diffractometer. The scattered x-rays were collected using a two-dimensional detector (Pilatus) enabling fast data acquisition. The data were recorded using a fixed x-ray incidence angle of 0.5°. Kiessig oscillations of the ultrathin ferroelectric layer prove the very good long-range quality of the latter.

For the atomically resolved characterization we used an aberration-corrected (Cs probe corrector) FEI TITAN 80-300 analytical scanning transmission electron microscope (STEM), allowing a spatial resolution of about 0.8 Å in the HREM and STEM mode as well. Applying a high angle annular dark field detector (HAADF) in the STEM mode, elastic, thermal diffuse scattering (TDS) events can be recorded. The intensity of these localized, incoherent scatter processes is proportional to Z^2^, and thus, the position of atom columns or individual atoms is imaged with a brightness related to their atomic number Z. This is usually referred to as Z-contrast technique.

The temperature dependent magnetoelectrical measurements were carried out inside a cryo-probing station containing a superconducting magnet with a maximum applicable magnetic field of 1 T. TMR curves were measured with a Keithley 2635 source meter by applying a voltage of 50 mV to the junctions and continuously ramping the magnetic field while measuring the tunnel current. Normalized TMR ratios were calculated using:


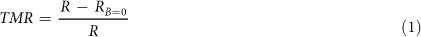


Here, R is the measured resistance, R_B=0_ the resistance at zero magnetic field and TMR the relative value in percent. To switch into the respective ferroelectric TER states, short (5 μs) voltage pulses of ±3 V have been applied. Proper electron tunneling was confirmed with fits using the Brinkman model^30^.

## Author Contributions

A.Q., I.F. and X.M. wrote the main manuscript text and A.Q. prepared all figures. A.Q., I.F., M.A. and D.H. conceived and designed the experiment. G.A. conducted SQUID measurements, E.P. did TEM investigations and P.F. and C.N. were responsible for the SXRD data. All authors reviewed the manuscript.

## Additional Information

**How to cite this article**: Quindeau, A. *et al*. Four-state ferroelectric spin-valve. *Sci. Rep.* **5**, 9749; doi: 10.1038/srep09749 (2015).

## Supplementary Material

Supplementary Information

## Figures and Tables

**Figure 1:**
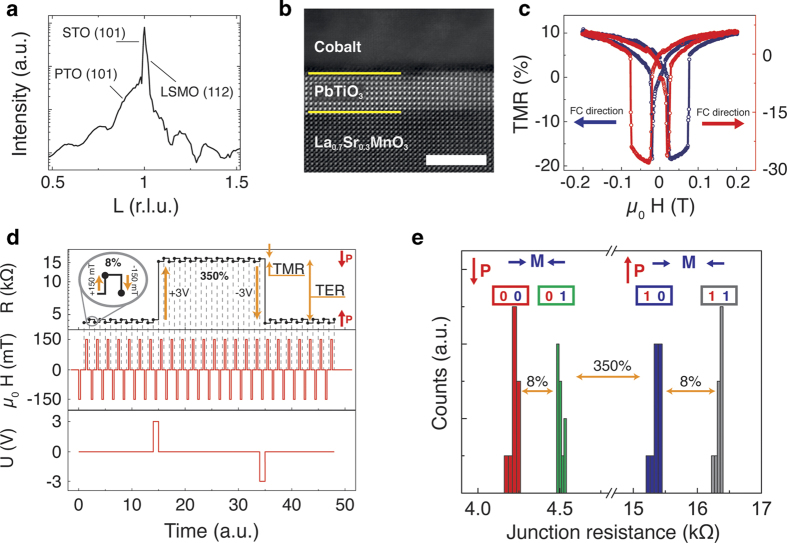
(**a**) SXRD data from (10 L) crystal truncation rods (CTRs). Reflections coming from STO, PTO and LSMO are indicated. (**b**) HAADF/STEM image of the tunnel junction, scale bar 5 nm. (**c**) TMR loops at 5 Kelvin of a LSMO/PTO/Co tunnel device, cooled down in a magnetic field (-800 mT for the blue, +800 mT for red curve, respectively). Exchange bias is visible on the crossing of the butterfly curves at non-zero magnetic field. (**d**) Resistance measured at full remanence (zero electric field, zero magnetic field) after the application of 150 mT, and ±3 V. (**e**) Event-counting histogram after 40 measurements showing the separation between the four resistive states.

**Figure 2:**
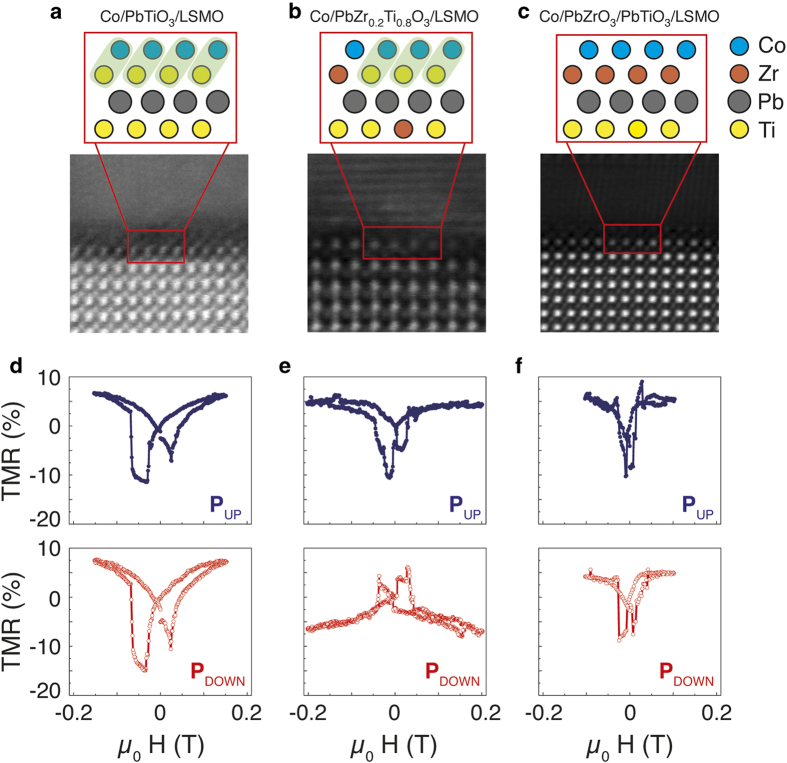
(**a**, **b**, **c**) Schematics and HAADF/STEM images of the FE-FM interfaces, illustrating the dilution of the titanium at the interfaces by zirconium for Co/PTO/LSMO, Co/PZT/LSMO and Co/PZO/PTO/LSMO, respectively. Shadowed atoms are those Co and Ti ones that are magnetically coupled. (**d**, **e**, **f**) TMR loops for ferroelectric polarization states pointing towards the LSMO electrode (red) and the cobalt electrode (blue).

**Figure 3:**
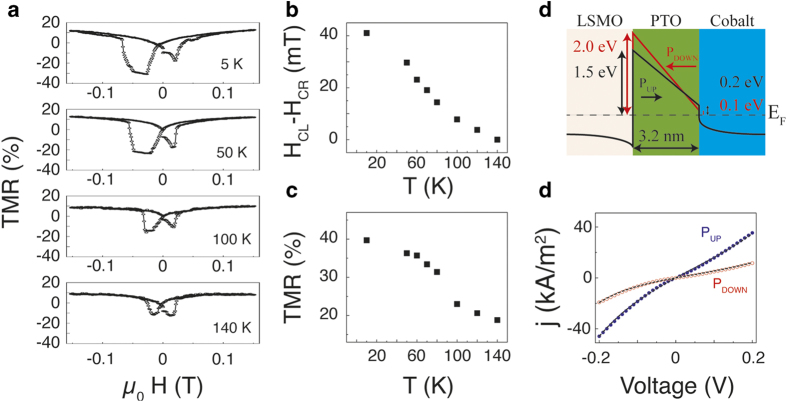
(**a**) TMR loops for different temperatures, showing a decrease in asymmetry until it becomes symmetric around 140 Kelvin. (**b**, **c**) Dependence of the asymmetry (coercive field “left” side (H_CL_) minus coercive field “right” side (H_CR_)) and TMR on temperature. (**d**) Schematics of the band structure according to the results obtained from the fitting parameters in e. (**e**) IV- characteristic curves after switching the ferroelectric polarization with electric pulses of 3 V and 100 μs. The blue and red points represent measurements for low and high resistance states of the respective junctions with ferroelectric polarization pointing towards and away from the cobalt electrode, respectively. The black dashed lines inside the figures are fits on the data with the Brinkman model. All measurements were performed at 5 Kelvin.
