# Supplementary material for: Four-state ferroelectric spin-valve
Source: Sci Rep. 2015 May 11;5:9749. doi: 10.1038/srep09749 (PMC4426701; doi:10.1038/srep09749)
Supplement: Supplementary Information [file srep09749-s1.doc]

Supplementary information on: Four-state ferroelectric spin-valve

*Andy Quindeau^1^,*, Ignasi Fina^1,2^,*, Xavi Marti^3,4^, Geanina Apachitei^2^, Pilar Ferrer^5^, Chris Nicklin^5^, Eckhard Pippel^1^, Dietrich Hesse^1^, Marin Alexe^1,2^,**

^1^Max Planck Institute of Microstructure Physics, D-06120 Halle, Germany

^2^Department of Physics, University of Warwick, Coventry CV4 7AL, United Kingdom

^3^Institute of Physics ASCR, v.v.i., Cukrovarnická 10, 162 53 Praha 6, Czech Republic,

^4^Centre d’Investigacions en Nanociencia i Nanotechnologia (ICN2), CSIC-ICN, Bellaterra 08193, Barcelona, Spain

^5^Diamond Light Source, Harwell Science and Innovation Campus, Chilton, Didcot OX11 0DE, United Kingdom

*Corresponding authors: A. Quindeau: [quindeau@mpi-halle.de](mailto:quindeau@mpi-halle.de)

I. Fina: ignasifinamartinez@gmail.com

M. Alexe: [M.Alexe@warwick.ac.uk](mailto:M.Alexe@warwick.ac.uk)

In Figure S1, we show the performed ferroelectric characterization of the ferroelectric layer of the samples characterized in the main manuscript (PTO/LSMO, PZT/LSMO, and PZO/PTO/LSMO) by means of DART-PFM spectroscopy (experimental details included below). In the figure the hysteretic 180° phase contrast reveals the expected ferroelectric behavior, although we observe an offset along the applied-voltage axis for the pure PTO case. The found voltage shift (commonly named ferroelectric imprint) can be expected since the tunnel barriers during the PFM measurements were not capped with the electrode material. The very different screening lengths and dielectric constants of the bottom electrode and the conductive tip lead to asymmetries in the polarization values for opposite directions^[SR1]^. The screening of surface charge by the Co top electrode would compensate the offset in the ferroelectric coercive fields as it can be seen on all the TER measurements performed.

Remarkably, the ferroelectric nature of the Co/PZO/PTO/LSMO sample could be demonstrated, even though the antiferroelectric (in bulk) PZO is capping the hetero structure. However, due to an already demonstrated phase transition for ultra thin PZO films less than 5 unit cells, PZO is anticipated to not comprise any antiferroelectric characteristics^[SR2]^.

Finally, the measured hysteresis loops indicate that the coercive fields of the films increase with increasing amount of zirconium at the surface, being the strongest for PZO-terminated PTO. This conclusion matches with the observation of increasing voltage that is needed to switch the junctions into opposite TER states.

Experimental details: PFM measurements were performed with a MFP-3D Asylum Research microscope. AppNano Co. cantilevers with Pt coating (ANSCM-PT) have been used. To achieve better sensitivity, the dual AC resonance tracking (DART) method was employed^[SR3,SR4]^. PFM voltage hysteresis loops were always performed at remanence, using an AC excitation signal of 100 mV and a dwell time of 100 ms. The quantification of the piezo coefficient using DART is difficult due to the simultaneous variation of measurement frequency and the variation of the maxima of the resonance amplitude while measuring; consequently the performed experiments have not been included here.

**Figure S1.** Phase signal of DART-PFM hysteresis loops for PTO/LSMO, PZT/LSMO and PZO/PTO/LSMO samples, performed at room temperature.

In Figure S2, we present the recorded M(H) loops measured at 5 K for PTO/LSMO (with respective thicknesses equal to the Co/PTO/LSMO sample of the manuscript) and Co/PTO/LSMO (characterized in the manuscript). From the figure and its comparison with Figure 1c in the manuscript, it can be inferred that the Co and LSMO coercive fields are in accordance with the ones measured by TMR loops. Interestingly, the loops recorded after FC conditions equal to those used in the performed TMR experiments, and thus do not reveal the presence of EB, which is another indicator that the found effect is confined to not more than several monolayers. M(H) loops performed with FC along different directions resulted in similar observations.

Because the thicknesses of the magnetic layers are equal in all samples, the shown magnetic data are representative for all of them.

Experimental details: Magnetic measurements were performed with a SQUID magnetometer of Quantum Design Co.

**Figure S2.** M(H) loops for LSMO/PTO and LSMO/PTO/Co samples, after FC procedure.

In Figure S3, the perpendicular-to-plane magnetoresistance of a LSMO/PZT/Cu tunnel junction is shown. The obtained ≈20% resistance variation, much larger than the magnetoresistance obtained in nominally equal bare LSMO films and similar to that measured in the junctions of the main text, has been ascribed to TAMR.^[SR5]^


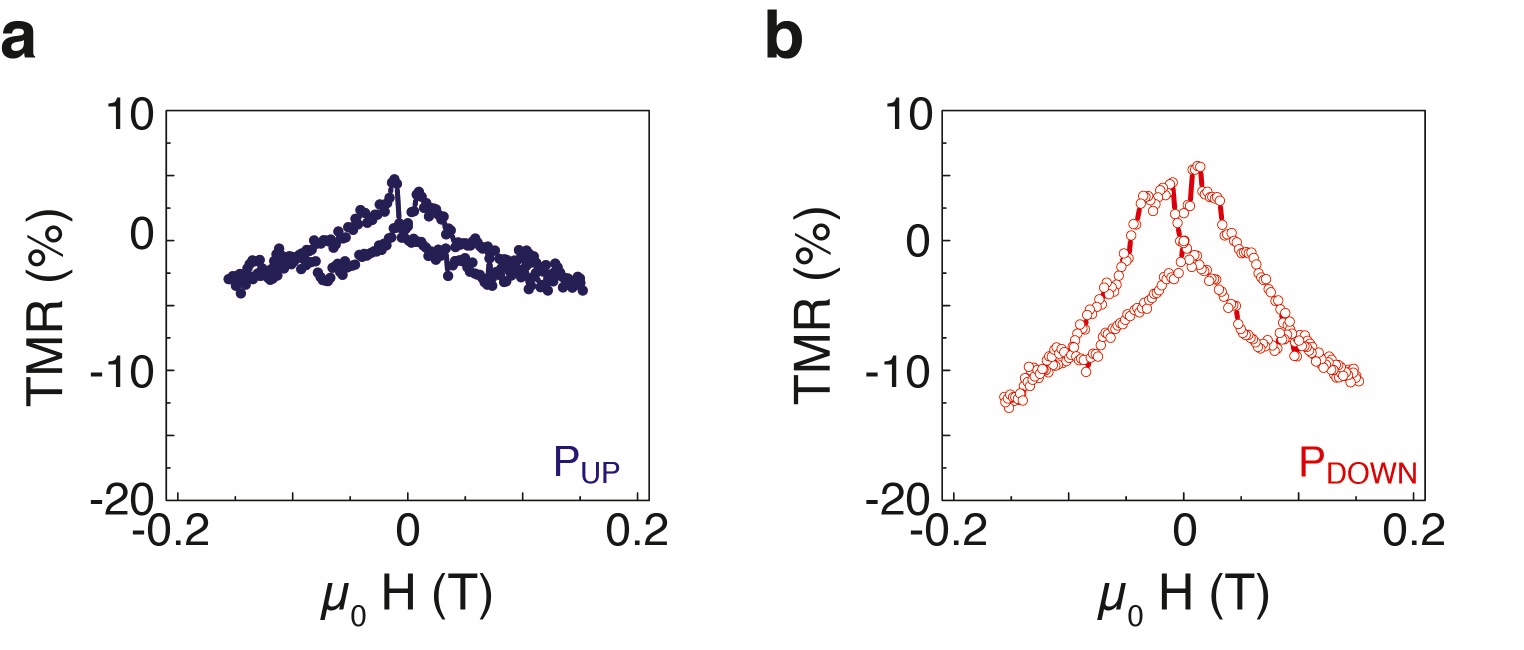


**Figure S3.** MR loops of a junction where ferromagnetic cobalt was replaced by non-ferromagnetic copper at the top electrode, for two ferroelectric polarization states P_UP_ (a) and P_DOWN_ (b),

In Figure S4 a,c, the 8 and 7.5 RHEED oscillations demonstrate the layer-by-layer growth mode for the respective number of monolayers of PTO and PZT for Co/PTO/LSMO and LSMO/PZT/Co samples, respectively. Figure S4 e shows the RHEED oscillation for PZO (ablation was stopped right at the intensity maximum of the first oscillation) resulting in a monolayer of PZO on top of a 7 unit cell thin PTO layer (RHEED oscillations not shown). The morphology of the three samples, characterized by AFM and displayed in Figure S4 b,d,f, resembles the step terraces of the STO substrate, also indicative of the layer-by-layer growth and the good quality of the films.


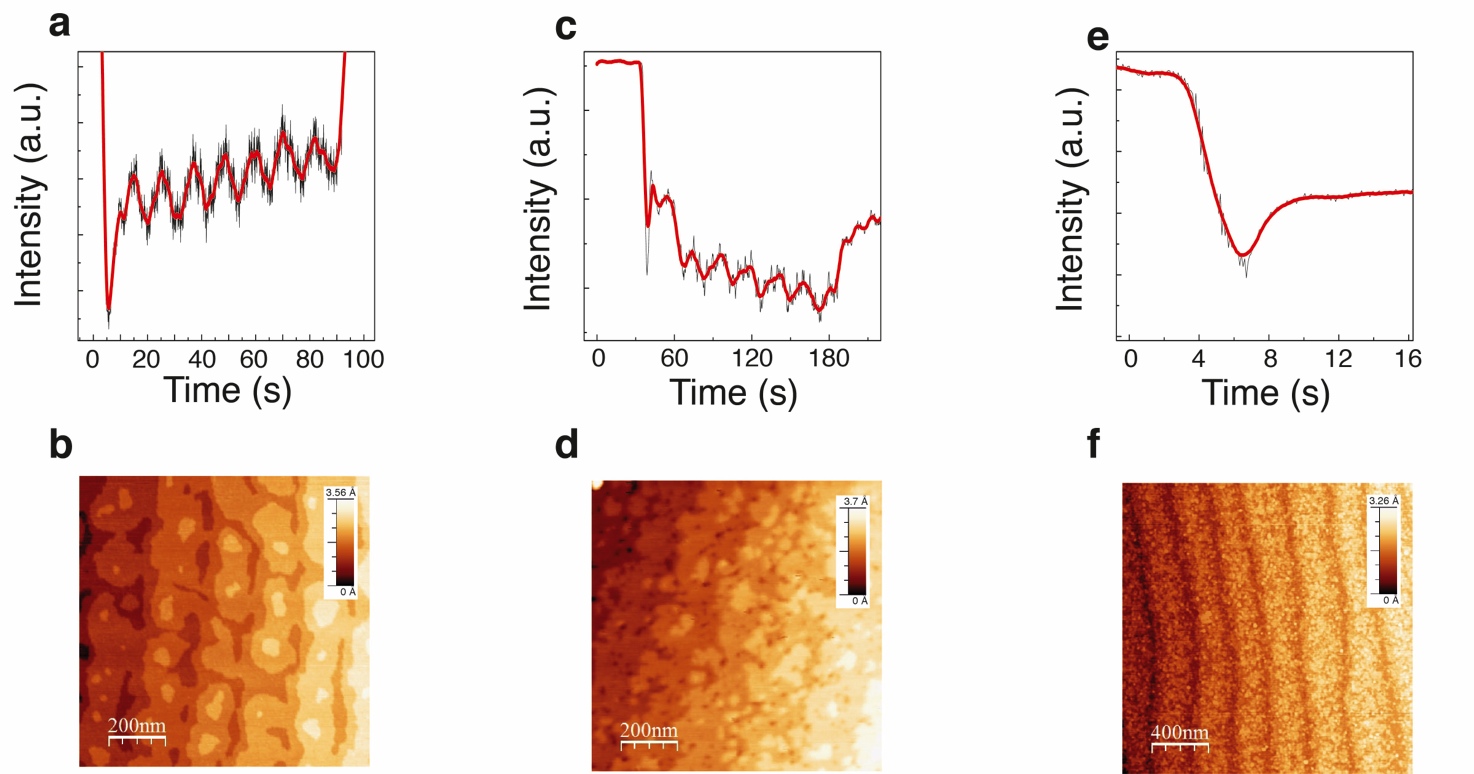


Figure S4. RHEED intensity oscillations for the grown (a) PTO-, (c) PZT- and (e) PZO barriers. Atomic force microscopy images of the ferroelectric barriers: PTO (b), PZT (d) and PTO/PZO (f),.

[SR1] Liu, Y., Lou, X., Bibes, M. & Dkhil, B. Effect of a built-in electric field in asymmetric ferroelectric tunnel junctions. *Phys. Rev. B* **88**, 024106 (2013).

[SR2] Boldyreva, K., Pintilie, L., Lotnyk, A., Misirlioglu, I. B., Alexe, M. & Hesse, D. Thickness-driven antiferroelectric-to-ferroelectric phase transition of thin PbZrO3 layers in epitaxial PbZrO_3_/Pb(Zr_0.8_Ti_0.2_)O_3_ multilayers. *Appl. Phys. Lett.* **91**, 122915 (2007).

[SR3] Rodriguez, B. J., Callahan, C., Kalinin, S. V. & Proksch, R. Dual-frequency resonance-tracking atomic force microscopy. *Nanotechnology* **18**, 475504 (2007).

[SR4] Rodriguez, B. J., Jesse, S., Seal, K., Balke, N., Kalinin, S. V. & Proksch, R. [Chapter 17] Scanning Probe Microscopy of Functional Materials, [Kalinin, S. V. & Gruverman, A.] (Springer, New York, 2011).

[SR5] Gould, C., Rüster, C., Jungwirth, T., Girgis, E., Schott, G. M., Giraud, R., Brunner, K., Schmidt, G. & Molenkamp,, L. W. Tunneling Anisotropic Magnetoresistance: A Spin-Valve-Like Tunnel Magnetoresistance Using a Single Magnetic Layer. *Phys. Rev. Lett.* **93**, 117203 (2004).
